# Videodialysis: a pilot experience of telecare for assisted peritoneal dialysis

**DOI:** 10.1007/s40620-019-00647-6

**Published:** 2019-09-16

**Authors:** Giusto Viglino, Loris Neri, Sara Barbieri, Catia Tortone

**Affiliations:** SOC Nefrologia e Dialisi, Ospedale San Lazzaro, Via Pierino Belli 26, 12051 Alba, CN Italy

**Keywords:** Peritoneal dialysis, Barrier to self care, Assisted peritoneal dialysis, Telemedicine, Patient empowerment

## Abstract

**Background:**

We report our experience with Videodialysis (VD), a new telemedicine system created in our Center to overcome physical, cognitive and psychological barriers to PD.

**Methods:**

We analyzed the technical and clinical care results of VD in the period from 01/01/2009 to 12/31/2018.

**Results:**

The VD components are: a Remote Station at the patient’s home (video camera, monitor, microphone, technological connectivity box), and a Control Station in the Center (PC with high resolution monitor, webcam, speakerphone) with software that manages 6 audio-video connections simultaneously as well as the Remote Station video cameras. In 2015 a second model of VD was designed to further improve ease of transport, installation, user interface, software functionality and connectivity. VD proved to be highly reliable during 21,000 connections, and easy to use by patients/caregivers without technological skills. During the observational period, 107 patients started PD; of these 77 had barriers to PD: in 15 we overcame the barriers by VD-Assisted PD and in 62 we used other modalities of Assisted PD. During a follow-up of 285 months on VD-Assisted, 5 patients died, 3 were transferred to HD (UFF; leakage; onset of barriers insurmountable with VD), 3 to traditional Assisted PD and 4 remained on VD-Assisted PD. Peritonitis incidence in VD-Assisted PD was 1/84.2 pt/mths, not significantly different to that of the patients not using VD. Sense of confidence was the aspect most highly-appreciated by VD-Assisted PD patients.

**Conclusions:**

VD-Assisted PD is a reliable, safe system which requires no technological know-how and it is easy to use when self-care is not possible due to physical, cognitive or psychological barriers.

**Electronic supplementary material:**

The online version of this article (10.1007/s40620-019-00647-6) contains supplementary material, which is available to authorized users.

## Background

As it happens with other self-care activities, a number of social, physical, cognitive and psychological barriers may limit the use of Peritoneal Dialysis (PD).

In such cases, it is necessary to resort to Assisted PD: Family Caregiver, Nurse at home, PD in Nursing Home.

The recent development of telemedicine has made it possible to pursue new models of remote care and treatment. Telemedicine has also been applied in PD, and though most of the experiences have related to data transfer and processing; the few that have involved its application to self-care patient follow-up have demonstrated improved monitoring and fewer visits to the Center [1, 2]. No experience has been reported, on the other hand, of the use of telemedicine in PD as a tool for overcoming barriers to self-care and improving patient empowerment.

From 2002 to 2008, in order to avoid drop-out to hemodialysis (HD) a Sony videoconferencing device was used as technological support to overcome the physical, cognitive and psychological barriers to Self-care Peritoneal Dialysis that had arisen in 7 patients on PD [3].

The first positive results achieved in this initial experience led us to devise a specially-designed system in order to create a “video caregiver” which would overcome barriers to self-care in PD. This system was called Videodialysis (VD).

VD was incorporated as an additional option within the traditional framework of Assisted PD provided in our Center (Family Caregiver–Nurse at Home–PD in Nursing Home), with the aim of extending the possibilities of using PD, and reducing the family care burden and recourse to nurses at home.

We report our experience with VD from 01/01/2009 to 12/31/2018, analyzing the following aspects: technological characteristics and evolution, indications and methods of use, clinical results and patient satisfaction.

## Materials and methods

The VD system used in this study was designed and built in our Center.

**Fig. 1:**
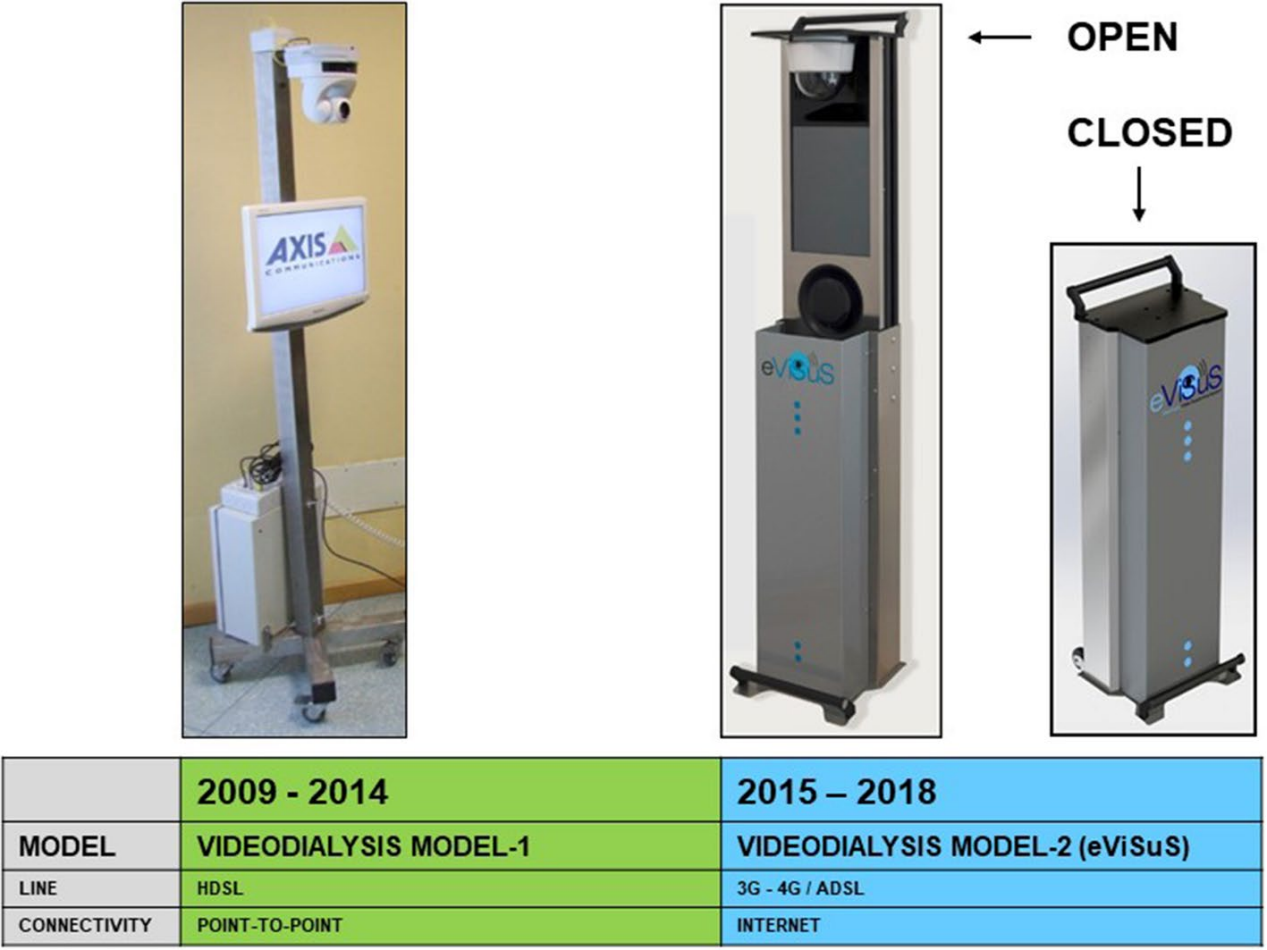
Evolution of the VD system used in our Center

During the observation, two VD models were developed: the first introduced in 2009 (VD-Model1) was improved technologically in 2015 (VD-Model2-eViSuS) (Fig. 1). The details of the technical specifications are reported in Supplementary Material—Part 1.

The VD-Assisted PD sessions were arranged for set times during the day so as to guarantee assistance from the Control Station during the CAPD exchanges, APD cycler preparation, connection and disconnection procedures. The way in which VD is used is reported in detail in Supplementary Material—Part 2.

Patients underwent clinical-psychosocial-aptitude assessment by the medical-nursing team during the pre-dialysis pathway, and if no absolute contraindications emerged, patients chose dialysis modality. If PD was chosen and it was considered that the barriers could be overcome with VD, patients were started on VD-Assisted PD. If not, recourse to a family-member caregiver was evaluated, with priority being given to the lowest possible impact on the household in terms of financial cost and quality of life. A similar assessment of the presence of barriers was also carried out for caregivers, assisting them with VD in the event of barriers which could be overcome using this support. Satisfaction with VD was evaluated by the choice to start using this type of Assisted PD and not to abandon it during the follow up. Furthermore, the opinions of all the patients assisted with VD during July–September 2015 (5 patients and 1 caregiver) were investigated more in depth by means of semi-structured interviews conducted by an appropriately-prepared nurse who was not from the Center. The method of choosing between dialysis treatment and the various Assisted PD options, and of evaluating satisfaction with VD-Assisted PD are described in detail in Supplementary Material—Part 3.

All incident patients between 01/01/2009 and 12/31/2018 were considered, examining the choice of treatment, the modality of Assisted PD and the types of barrier.

The clinical results of VD were evaluated by comparing peritonitis in self-care patients and patients with a family-member/live-in caregiver and analyzing technical failure and drop-out to HD.

The study was approved by the Inter Hospital Ethics Committee A.O. “S. Croce e Carle” di Cuneo e AA.SS.LL. Cuneo 1, Cuneo 2, Asti (Prot.18041-02/18).

## Results

**Fig. 2:**
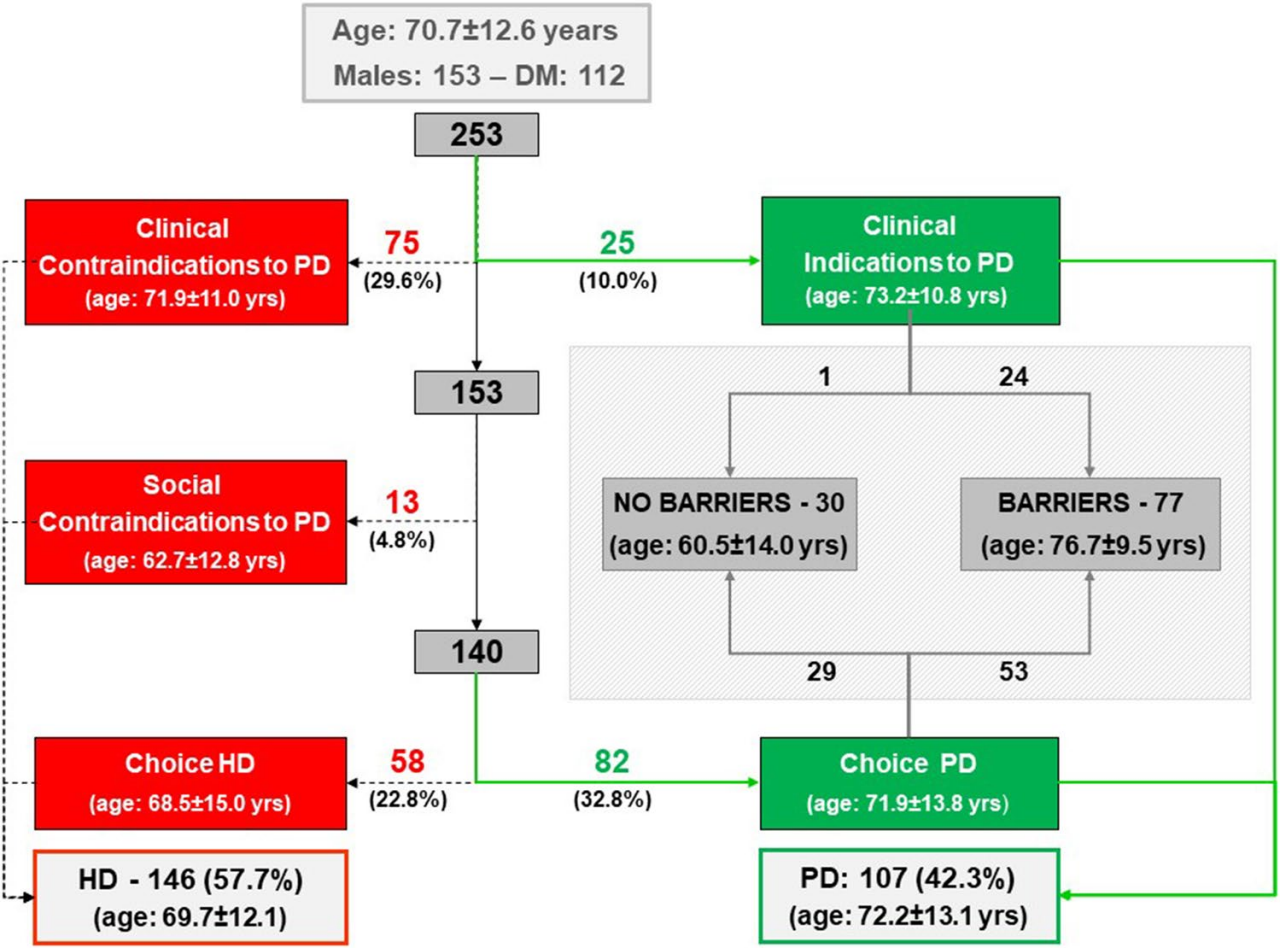
Choice of dialysis treatment in incident patients on dialysis at our Center in the period 01/01/2009–12/31/2018

**Fig. 3:**
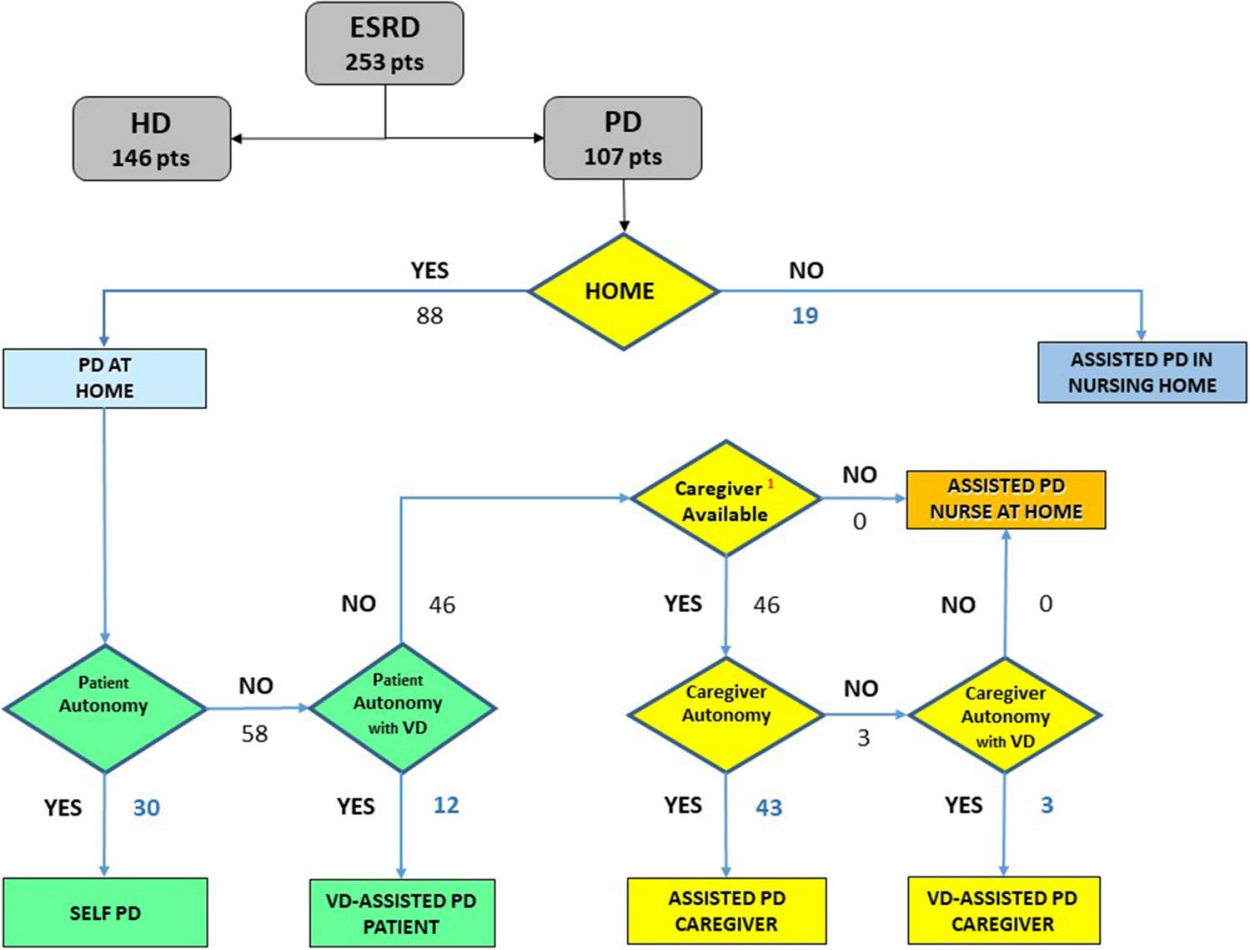
Flow chart and results of the choice between self PD and the various methods of Assisted PD (Assisted PD VD Patient, Assisted PD Caregiver, Assisted PD VD Caregiver, Assisted PD Nurse at Home, Assisted PD in Nursing Home). ^1^The choice of family caregiver must prioritize the lowest possible financial and quality-of-life impact on the household

The results of the choice of dialysis treatment and of the various methods of Assisted PD in the observation period are reported in Figs. 2 and 3 respectively.

Of the 107 patients who started on PD, 30 were independent and 77 had barriers preventing self-management. The characteristics of the independent patients and those on the various forms of Assisted PD are reported in Table 1.

**Table 1:** Number and characteristics of patients divided by Self PD and the different types of Assisted PD

PD modality	Number	Age	Male	DM
Self PD	30	60.5 ± 14.0	23	8
VD-assisted PD (patient)	12	73.7 ± 9.3	6	7
Assisted PD caregiver (spouse)	13	66.4 ± 9.7	9	8
Assisted PD caregiver (son/daughter)	19	80.4 ± 7.0	7	5
Assisted PD caregiver (live-in carer)	11	80.1 ± 7.2	6	7
VD-assisted PD caregiver (spouse)	3	77.1 ± 2.5	3	2
Assisted PD in nursing home	19	80.0 ± 8.4	9	8
Total	107	72.2 ± 13.1	63	45

All the 12 patients (6 without a caregiver) with barriers which could be overcome with VD chose to be assisted directly by the video caregiver in the performance of the dialysis procedures. In 3 other cases the “video caregiver” assisted the patients’ wives (age: 77.3 ± 1.5 years). The barriers overcome by VD are reported in Table 2.

**Table 2:** Barriers to self-management in the 12 patients and in 3 caregivers on VD

Barriers	VD-assisted PD patients	VD-assisted PD caregivers	VD-assisted PD total (%)
Number	12	3	15
Physical			
Sight	6	1	7 (15.2)
Hearing	2	0	2 (4.3)
Manual dexterity	2	0	2 (4.3)
Physical strength	0	0	0 (0.0)
Cognitive			
Cognitive deficit	2	0	2 (4.3)
Understanding/memory/language	5	2	7 (15.2)
Compliance	3	0	3 (6.5)
Psychological			
Anxiety/fear of self-management	10	2	12 (26.1)
Depression	10	1	11 (23.9)
Total	40	6	46 (100.0)

Average follow-up for the 15 VD patients was 19.0 ± 12.9 months. During the 21,000 follow-up connections, VD-Assisted PD proved to be highly reliable and easy to use by personnel and patients or their caregivers, without any special technological skills being required.

During a follow-up of 1869 patient-months, 34 episodes of peritonitis were recorded: 1 every 84.2 months in VD-Assisted PD patients, 1 every 62.6 months with a family member/live-in carer patients, 1 every 45.2 months in self-care patients. Time free from first peritonitis was not different between the three groups.

During the follow-up of the 15 VD patients, 3 (20.0%) were transferred to HD (1 UFF, 1 pleuro-peritoneal communication, 1 onset of barriers insurmountable with VD), 3 (20.0%) switched to other modalities of Assisted PD (2 live-in carer, 1 family caregiver) due to a deterioration in clinical condition and barriers to the method, 1 patient (6.7%) had a transplant.

No patients/caregivers chose to drop out from VD-Assisted PD. All the patients interviewed expressed a favorable opinion of VD as a tool capable of instilling confidence; half of them also appreciated that it enabled them to stay independent, and another half to perform the dialysis at home. In 50% of the interviewees, the need to be subject to pre-arranged times for the connections and the performance of the dialysis procedures was seen as the only limiting factor.

During the course of the follow-up, in 17 of the 92 patients on PD without VD the need for drop-out to HD was avoided in 3 patients (17.6%) thanks to recourse to VD-Assisted PD: 2 cases of self-care patients with reduced compliance and a high number of episodes of peritonitis, and 1 case of a family-member caregiver no longer being available.

## Discussion

This remote management system represents a unique, innovative experience from a technological point of view for overcoming barriers to self-management of PD.

The high number of connections performed (over 21.000) proved the system to be highly reliable and easy to use by personnel, patients and their caregivers, without any special technological skills being required.

With the introduction of VD-Model2-eViSuS, the system’s usability and flexibility were improved considerably thanks to its ease of transport, installation without the assistance of technical personnel, and mobile internet connection without the need for dedicated phone lines.

From this point of view, this experience is technological far more advanced than the system used by Gallar [1], which is comparable to the Sony videoconferencing device used initially in our experience [3].

Finally, the mobile internet connection has also allowed for considerable cost savings in managing connectivity to be made compared to ADSL/SHADSL lines.

The management of CAPD/APD with VD was made possible through the reorganization of nursing work and precise standardization of dialysis procedures and assessment of clinical issues. The application of this organizational model showed that one nurse can manage up to 6 patients at the same time during a session of VD lasting approximately 1 h.

VD allowed for a 6% increase in the use of PD in the incident and particularly elderly population, corresponding to 14% of those starting on PD. From a clinical and care point of view, with VD it was possible to create a “video caregiver” as a way of overcoming barriers to PD in patients and/or their caregivers, especially with cognitive and psychological barriers which are particularly frequent in the elderly population [4, 5].

This creation of a “video caregiver” also made it possible not to refer to a family member or professional caregiver, which involves significant social [6] and economic [7, 8] costs.

If it is necessary to have recourse to a family-member caregiver, with VD it is also possible to use family members with barriers like elderly partners, thereby avoiding the use of younger members of the family.

As regards economic costs, the comparison should be made with Assisted PD carried out by a nurse at home [7, 8]. Compared to the latter, VD allows for considerable savings associated with the elimination of travelling costs and the fact that up to 6 patients can be followed at the same time instead of just 1. Conversely, VD entails higher costs relating to technology and connectivity. In this regard, at the time of the study the cost of VD could not yet be determined with precision, as the system was still evolving technologically. In general, however, it can be suggested that this type of cost will be reduced on the reaching of sufficient economies of scale. This important point has been the subject of a multi-center study in progress.

The peritonitis rate in patients on VD was low and not significantly different to that of self-care or other forms of Assisted PD patients, demonstrating the clinical safety and effectiveness of the VD system.

VD allowed a 17.6% reduction in drop out from PD to HD due to reduced compliance or lack of availability of a caregiver. This is particularly significant if it is considered that in the 2016 PD Census in Italy choice or impossibility to continue PD due to these aspects was the second cause of drop out (22.2%) after peritonitis.

Satisfaction with VD is shown not only by the fact that all the 12 patients for whom it was indicated accepted its use, but also by the fact that no patients or caregivers chose to change dialysis modality or Assisted PD modality during the follow up.

Furthermore, all the interviewees express their appreciation of VD as a support tool at home that allows them to feel confident in performing the dialysis procedures by themselves and remain independent, overcoming one of the main barriers to PD in the elderly population: the “fear of failure”. This barrier was present in 80% of cases of use of VD. Other studies [9] show how the choice not to do PD in a nephrology center is conditioned by the fear patients have of not being able to manage the method by themselves or the feeling of being alone.

Using VD for training could help both aspects: to make patients more confident about PD, and to improve the quality of training allowing better customization, time optimization and checking of all the steps [10]. From this point of view, VD could be a fundamental and very useful tool to grow the use of PD therapy, increasing the number of eligible patients and reducing drop-out due to poor compliance with dialysis procedures.

## Conclusions

In conclusion, from a technological point of view, VD is a reliable, safe system which is easy to use without requiring any technological knowhow.

In our experience, VD delivered a “video caregiver” with which PD can be started or continued in cases in which self-management of the technique by the patient or family caregiver is not possible due to the presence of physical, cognitive or psychological barriers.

By furthering patient empowerment in the management of their chronic disease, VD avoids recourse to forms of care which have a higher social (Family Caregiver) or financial (Nurse at Home) costs.

We believe that VD can be a fundamental and useful tool for increasing the eligibility of patients for PD therapy and reducing drop-out from the method. However, the applicability of VD on a wide scale and to other areas such as training or improving therapy compliance requires further study.

## Electronic supplementary material

Below is the link to the electronic supplementary material.

Supplementary material 1 (PDF 235 kb)
