# Supplementary material for: Videodialysis: a pilot experience of telecare for assisted peritoneal dialysis
Source: J Nephrol. 2019 Sep 16;33(1):177–82. doi: 10.1007/s40620-019-00647-6 (PMC7007422; doi:10.1007/s40620-019-00647-6)
Supplement: Supplementary file 1 — Supplementary material 1 (PDF 235 kb) [file 40620_2019_647_MOESM1_ESM.pdf]

SUPPLEMENTARY MATERIAL

PART 1: TECHNICAL CHARACTERISTICS AND EVOLUTION OF VIDEODIALYSIS.

The VD system used in this study was designed and built in our Center.
During the observation, two VD models were developed: the first introduced in 2009 (VD-Model1) was improved technologically in 2015 (VD-Model2-eViSuS) (**Figure 1**).
VD-Model1 was created on the basis of the experience acquired previously between 2002 and 2008 using the Sony videoconferencing device.
VD-Model2-eViSuS represents a technological evolution of the previous model in terms of both hardware and software, designed to increase the usability and flexibility of the System, with particular attention being paid to ease of transport, installation, user interface, software functionality and connectivity.

The basic components of the VD system are the Remote Station at the patient's home, the Control Station in the Center and the system connecting the two stations.
Pictures of the Remote Station are shown in **Figure 2**, along with the characteristics of the VD-Model1 and VD-Model2-eViSuS used in this study.
The Remote Station is composed of a video camera, a monitor, a microphone and a technological box containing the power supply and the electronics for connecting to the phone network. In VD Model2-eViSuS, the Remote Station was improved by introducing a self-contained structure which makes it easy to transport and activate at home without technical assistance.

The Control Station is composed of a webcam, a hands-free phone, a high resolution monitor and a personal computer with software installed which enables the connection of up to a maximum of 6 patients at the same time. By clicking the mouse on the different display windows it is possible to switch the audio between one Remote Station and another. The Remote Station video camera movement and zoom is controlled by moving the mouse in the Control Station display windows.
Compared to VD-Model1, the software installed on VD-Model2-eViSuS has a switch and pointer which make controlling the dialysis procedures more precise and efficient; the new software also enables the taking and storage of high resolution photographic images.

The connection between the two stations allows for real-time, high-quality audio-video transmission: the pictures taken by the Remote Station video camera are shown on the Control Station monitor, while the Control Station webcam pictures appear on the Remote Station monitor. Voice communication between the two stations is by speaker phone.
The connection in VD-Model1 was point-to-point using ADSL/SHDSL lines, whereas in VD-Model2-eViSuS the connectivity has been completely changed to create a network via internet to which the Remote Station and the Control Station can be connected by both landline and mobile.

Governance of the VD-Model2-eViSuS system is tasked to a Cloud Control Center, which is composed of a Central Cloud Server on which the software is installed to manage the system's functions: connecting the Remote Stations with the Control Station, online monitoring and support for the whole network, transmission of information (voice/images) using protected protocols, and enabling access to Remote Stations from other PCs and tablets as well as the Control Station.

PART 2: METHOD OF USE OF VIDEODIALYSIS

The method of use of the VD was standardized so that all nursing staff could follow patients and caregivers uniformly step by step during the following phases: multi-user connection, acquisition and recording of dialysis parameters, performance of the CAPD or APD dialysis procedure, and filling in of the dialysis sheets. The VD sessions were also used for exit site care, assessing any dialysis and/or clinical issues, and checking adherence to the pharmacological and/or dietary therapy.

The dialysis procedure with VD as "video caregiver" for nurse-assisted PD takes place as follows: connection, acquisition of the dialysis parameters and preparation of the material, performance of the procedure and filling in of the dialysis sheet.

- The connection requires activation of the system by the patient following advance notice given by the Center by phone; it takes place at set times previously agreed with each patient on the basis of the modality (CAPD / APD) and dialysis prescription (number of exchanges and daily or weekly sessions).

  Thanks to the multi-user function, during a single VD session lasting around 60 minutes the nurse is able to follow up to 6 patients in performing the dialysis procedures at the same time. The patients are supported by the VD in all the CAPD exchanges or APD dialysis procedures. The assistance is active every day of the year except Sundays and public holidays when the patient does not perform the dialysis treatment; if dialysis is required on those days, it is performed by a self-sufficient family caregiver. This method was used in 8 of the 15 patients on VD.
- When the connection has been activated, the dialysis parameters (weight, blood pressure, heart rate) are acquired in real time, before the first exchange of the day in the case of CAPD, and before the evening connection in the case of APD. Prior to each CAPD exchange or APD connection, the suitability of the material already set up by the patient or caregiver and the type of bags used are checked.
- The performance of the dialysis procedure (CAPD: exchange – APD: preparation, connection and disconnection) is guided/controlled remotely by the nurse from the Center.
- The type of bags used is recorded on the dialysis sheet, as are the time of the dialysis and the dialysis parameters (volume out and volume in, UF) at the end of the CAPD exchange and on the APD disconnection.

Furthermore, the connection makes it possible to check: the functionality of the catheter and the condition of the exit site, guiding the patient in medication; the presence/absence of any lower limb edemas; the appearance of the drained dialysate with the possibility to confirm turbidity by means of Cytur-test if necessary; glycemia in the case of diabetic patients; peripheral oximetry in the event of patients suffering from chronic respiratory failure.

In the event of any issues or situations that require more in-depth clinical evaluation, the nurse can ask to consult the nephrologist in the Dialysis Center, who can carry out a televisit with adjustment of the pharmacological or dialysis therapy.

PART 3: CHOICE OF DIALYSIS TREATMENT AND BETWEEN THE VARIOUS ASSISTED PD OPTIONS

The choice of dialysis treatment in our Center follows a similar process to the one described by Peter Blake (***Blake PG, Quinn RR, Oliver MJ.*** *Peritoneal dialysis and the process of modality selection. Perit Dial Int 2013; 33: 233-41*) which excludes patients with absolute, clinical or social-environmental contraindications to PD.

During the pre-dialysis pathway, patients eligible for PD are evaluated by a dedicated medical-nursing team during clinic and home visits for the presence of any physical, cognitive and psychological barriers to self-care. The evaluation was carried out following the indications of M. Gordon's theory (***Marjory Gordon*** *- Manual of Nursing Diagnosis - 2016*) using the following models: activity–exercise, cognitive-perceptive, health perception and management, tolerance to stress, perception and concept of self. These models were investigated and integrated with specific assessments more closely-related to the dialysis treatment.
The physical barriers of patients/caregivers are evaluated by means of general tests such as ADL/IADL, and above-all specific "in vitro" simulation of the dialysis procedure.

The cognitive (attention, understanding, memory, language, compliance) and psychological (anxiety, fear of self-care, depression) barriers are evaluated on the basis of the elements acquired during meetings with the patients and their family members, while certain specific tests such as MMSE and BDI-II are used in particular cases.

If there are any barriers, the different modalities of Assisted PD are then evaluated in relation to the care commitment required: Family Caregiver, Nurse at Home, PD in Nursing Home.
As regards Family Caregivers, the first option is to use the caregiver with the lowest economic and social impact. PD in Nursing Home is also taken into consideration for patients who are already in a Nursing Home or who need to be admitted to a Nursing Home on clinical-care grounds irrespective of the dialysis.

Within this traditional Assisted PD framework, the possibility of VD-Assisted PD is offered to patients/caregivers with barriers which could be overcome using VD in order to broaden the range of possible uses of PD and reduce the family burden and recourse to Nurses at Home.

Appreciation of VD-Assisted PD is shown initially by patients on their choice of dialysis treatment (HD or PD) and the various modalities of Assisted PD. Subsequently patients always have the possibility of changing dialysis treatment, or of choosing another Assisted PD options.
Between July and September 2015, the opinions of all the patients in treatment at that time with VD (5 patients and 1 caregiver) were investigated more in depth by means of semi-structured interviews conducted by an appropriately-prepared nurse who was not from the Center. In these interviews the patients talked freely about their experience with VD and how it affected their life, describing the positive and negative aspects. The script of each interview, which were all recorded and then transcribed, was then examined using A. Giorgi's phenomenological method and content analysis (suitable for a reduced cohort size) in order to assess the general appreciation of VD and identify the positive and negative aspects expressed by the patient (***1. Sadala ML, Bruzos GA, Pereira ER, Bucuvic EM.*** *Patients' experiences of peritoneal dialysis at home: a phenomenological approach. Rev Lat Am Enfermagem 2012; 20: 68-75* – ***2. Jhaveri D, Larkins S, Sabesan S.*** *Telestroke, tele-oncology and teledialysis: a systematic review to analyse the outcomes of active therapies delivered with telemedicine support. J Telemed Telecare 2015; 21:181–8*).

The results of this model of Assisted PD applied to incident patients in our Center between 2009 and 2018 are reported in detail below.

1. Choice of dialysis modality (Figure 2)

Of the 253 incident patients in the period considered, 75 had clinical contraindications to PD, represented by: in 39 an inaccessible abdomen (irreparable hernias, abdominal wall hernias, previous operations and adherences, stomia), in 8 a high risk of infection (previous diverticulitis, Crohn's

disease), in 5 immunodepression, in 12 pathological obesity, in 6 active neoplasms and in 5 other causes (respiratory failure, TBC, allergy to povidone-iodine).

In addition to these patients, a further 13 had absolute social-environmental contraindications to PD which made it impractical (no fixed address, degraded environment, psychiatric issues or problems of alcoholism or drug dependency).

In our experience, the patients eligible for PD were therefore 165 (65.2%), 58 of whom chose HD. The physical, cognitive and psychological barriers with different Assisted PD options were evaluated in the remaining 107 patients, 25 of whom had clinical indications to PD.

2. Choice of Assisted PD modality (Figure 3)

Of the 107 patients who chose PD, 19 were in residential care facilities due to the serious nature of their clinical conditions, and were not capable of self-care dialysis; in these cases, Assisted PD in Nursing Home was used.

The presence of barriers was evaluated in the remaining 88 patients on PD at home, with 30 being fully independent, and the remaining 58 having physical, cognitive and psychological barriers to self-care PD.

Based on the medical-nursing assessment, the barriers could be overcome using VD in 12 patients, including 6 without a caregiver: the details of the surmountable barriers are given in **Table II**. In particular, as regards the physical barriers, the problems of sight and manual dexterity present in 6 and 2 cases respectively were not sufficiently serious so as to compromise the possibility of performing the dialysis procedures: any errors relating to these barriers can be avoided, detected and corrected using VD. Problems of hearing in 2 cases were overcome by setting the volume on the device at a suitable level; there were no cases of physical strength issues.

With regard to the cognitive and psychological barriers present in more than 80% of the patients on VD, this system proves to be particularly effective, as is shown by the feeling of reassurance expressed by the patients in the interviews.

Barriers were present in the remaining 46 patients which could not be overcome with the use of VD. For these patients it was necessary to have recourse to a caregiver, who was chosen subsequently from among partners, children/children-in-law, live-in carers so as to limit the social and economic costs of the dialysis treatment.

In 16 of these patients, the caregiver was the partner: self-sufficient in 13 cases and with barriers which could be overcome using VD in the other 3.

Of the remaining 30 cases in which the partner was absent (21 patients) or had barriers which could not be overcome for the management of the PD (9 patients), the caregiver was found in 19 among their children. In the remaining 11 cases in which children were absent (3 patients) or not available (8 patients), the caregiver was identified in a live-in carer.

In no case was it necessary to have recourse to a Nurse at Home.
